# A case of osteomalacia due to deranged mineral balance caused by saccharated ferric oxide and short-bowel syndrome: A case report

**DOI:** 10.1097/MD.0000000000008147

**Published:** 2017-09-29

**Authors:** Hiroshi Nomoto, Hideaki Miyoshi, Akinobu Nakamura, So Nagai, Naoyuki Kitao, Chikara Shimizu, Tatsuya Atsumi

**Affiliations:** a Department of Rheumatology, Endocrinology and Nephrology, Faculty of Medicine and Graduate School of Medicine, Hokkaido University; b Division of Laboratory and Transfusion Medicine, Hokkaido University Hospital, Sapporo, Japan.

**Keywords:** fibroblast growth factor 23, mineral imbalance, osteomalacia, saccharated ferric oxide, short-bowel syndrome

## Abstract

**Rationale:**

Saccharated ferric oxide has been shown to lead to elevation of fibroblast growth factor 23, hypophosphatemia, and, consequently, osteomalacia. Moreover, mineral imbalance is often observed in patients with short-bowel syndrome to some degree.

**Patient concerns:**

A 62-year-old woman with short-bowel syndrome related with multiple resections of small intestines due to Crohn disease received regular intravenous administration of saccharated ferric oxide. Over the course of treatment, she was diagnosed with tetany, which was attributed to hypocalcemia. Additional assessments of the patient revealed not only hypocalcemia, but also hypophosphatemia, hypomagnesemia, osteomalacia, and a high concentration of fibroblast growth factor 23 (314 pg/mL).

**Diagnoses:**

We diagnosed her with mineral imbalance-induced osteomalacia due to saccharated ferric oxide and short-bowel syndrome.

**Interventions:**

Magnesium replacement therapy and discontinuation of saccharated ferric oxide alone.

**Outcomes:**

These treatments were able to normalize her serum mineral levels and increase her bone mineral density.

**Lessons:**

This case suggests that adequate evaluation of serum minerals, including phosphate and magnesium, during saccharated ferric oxide administration may be necessary, especially in patients with short-bowel syndrome.

## 1 Introduction

It has been reported that, in cases of short-bowel syndrome caused by extensive intestinal resection, deficiencies in water content, nutrients, electrolytes, and minor elements can occur because of malabsorption by the intestine. Abnormalities in clinical features and laboratory values for serum calcium, phosphate, and magnesium may be observed, depending on the function and length of the residual small intestine. However, serum mineral balances are tightly mediated by biological mechanisms such as hormonal and humoral factors. A humoral factor, fibroblast growth factor 23 (FGF23), which is produced and secreted from osteocytes, has been identified as the factor responsible for autosomal-dominant hypophosphatemic rickets/osteomalacia^[1]^ and the cause of tumor-induced rickets/osteomalacia.^[2]^ It has been demonstrated that FGF23 plays an important role in keeping phosphorus metabolism in balance by inhibiting phosphate reabsorption, both in the proximal tubule and the bowel.^[3]^ However, in recent studies, hypophosphatemia caused by intravenous administration of saccharated ferric oxide (SFO) was accompanied by inappropriate elevation of FGF23.^[4,5]^

We report a patient who developed several mineral imbalances caused by intravenous administration of SFO based on short-bowel syndrome and discuss the intricate balance of hormonal regulatory mechanisms.

## 2 Case presentation

The patient was a 62-year-old woman with a 24-year history of Crohn disease who had undergone multiple intestinal resections. Although gastrointestinal symptoms were recently minimal with administration of anti-TNF-α antibody and antidiarrheal drugs, she had made frequent trips to the hospital and received regular SFO administration to treat anemia. Over the course of treatment, she experienced numbness of her legs and was diagnosed with tetany due to hypocalcemia (serum calcium level approximately 6.0 mg/dL). Oral or intravenous calcium replacement therapy temporarily improved both her serum calcium level and numbness, but the patient's tetany and hypocalcemia recurred frequently and she had to be rehospitalized many times. She was then referred to our hospital for a further examination and treatment of hypocalcemia.

**Figure 1:**
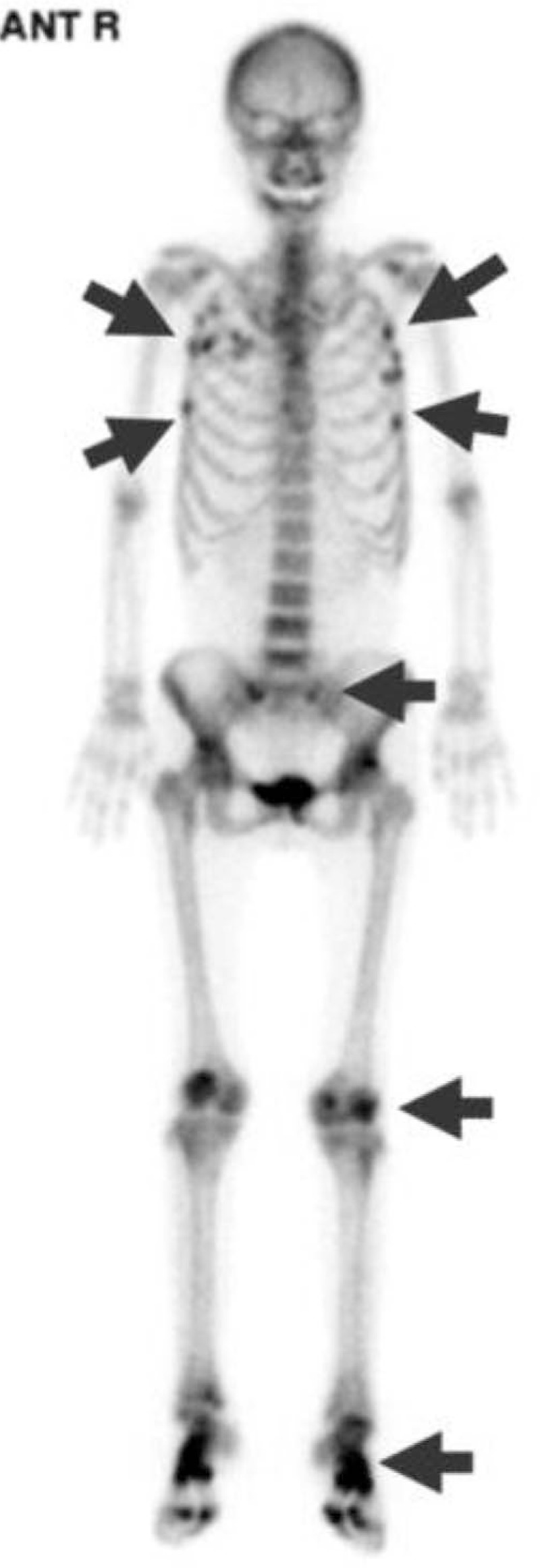
Bone scintigraphy. Bone technetium-99m methylene diphosphonate bone scan shows increased uptake (arrows) in the ribs, vertebrae, sacroiliac joints, knee joints, and ankle joints, suggesting multiple bone fractures and osteomalacia.

**Figure 2:**
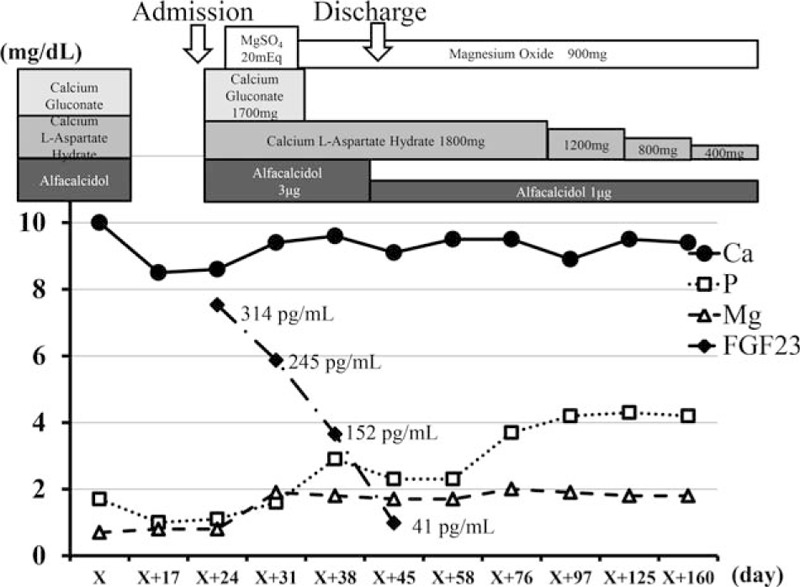
Clinical course. On admission, all supplemental treatment had been discontinued. However, because serum mineral levels could not be maintained without calcium supplements and alfacalcidol, administration of these drugs was resumed. Serum calcium levels normalized with intravenous and oral magnesium replacement therapy. Discontinuation of saccharated ferric oxide resulted in normalization of serum phosphate concentration accompanied by gradual reduction of FGF23. Following this, multiple serum mineral imbalances normalized despite reductions in calcium and vitamin D replacement. Ca = calcium, FGF23 = fibroblast growth factor 23, Mg = magnesium, MgSO_4_ = magnesium sulphate, P = phosphate.

On admission, her height was 152 cm (decreased by a few centimeters over the past few years) and her body mass index was 12.7 kg/m^2^. She had severe kyphosis. She had smoked 10 cigarettes per day for 42 years, did not consume alcohol regularly, and had no allergies. Family history was negative for electrolyte and mineral disorders. She showed malnutrition and laboratory analysis was remarkable for a low serum albumin level of 2.3 g/dL. Serum mineral concentrations were as follows: adjusted serum calcium 8.6 mg/dL after correction with calcium gluconate, calcium L-aspartate hydrate, and alfacalcidol; potassium 3.3 mEq/L; phosphate 1.1 mg/dL; and magnesium 0.8 mg/dL (Table 1). Bone scintigraphy revealed multiple uptakes in the ribs, spine, pelvis, and leg joints (Fig. 1). Because serum mineral levels could not be maintained without calcium supplements and alfacalcidol, as shown in Fig. 2, administration of these drugs was resumed.

**Table 1:**
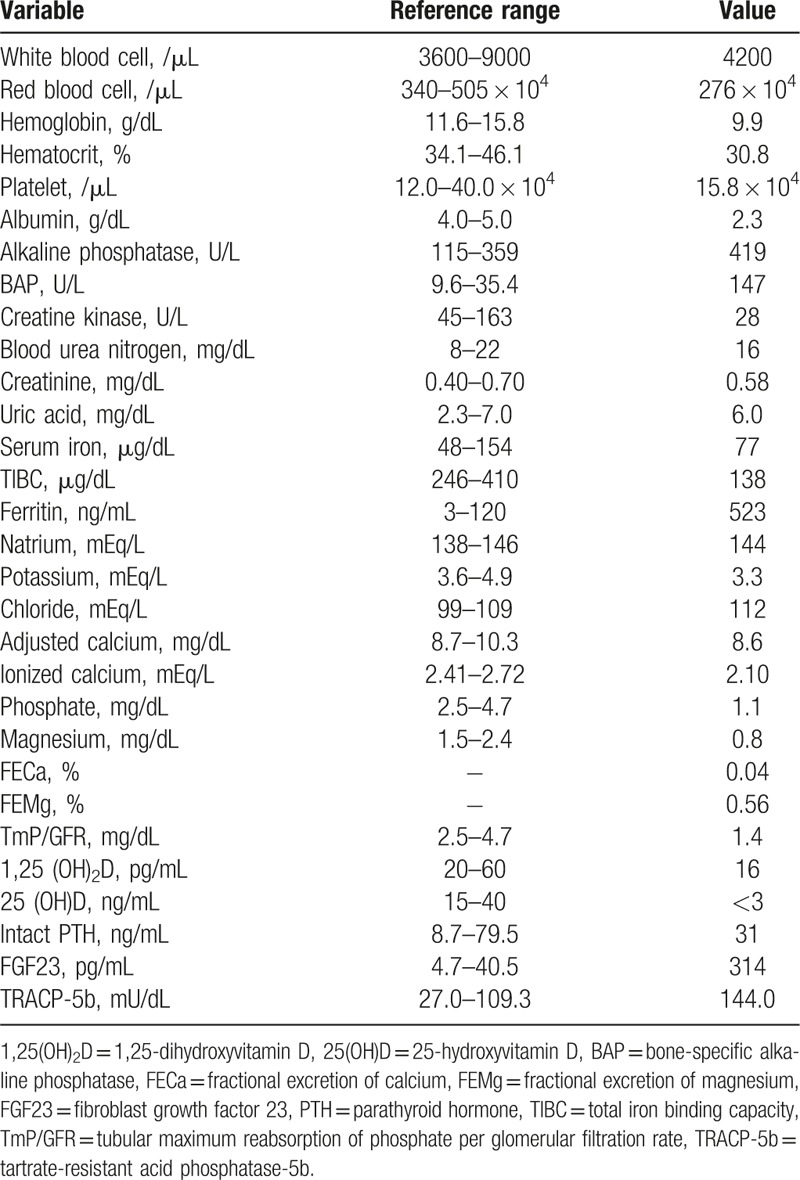
Laboratory tests on admission.

Assuming that one cause of her multiple mineral imbalances was hypomagnesemia, we first attempted to treat magnesium deficiency. Intravenous magnesium replacement followed by oral administration improved the deficiencies, not only of magnesium, but also partially of calcium and phosphate. Further investigation revealed that the level of FGF23 was extremely high (314 pg/mL); we then became aware of the patient's history of regular administration of intravenous SFO prior to our consultation. Because it has been reported that SFO can lead to hypophosphatemia via elevation of FGF23, we discontinued the SFO. Following this, the patient's FGF23 concentration gradually decreased, and multiple serum imbalances normalized despite reducing calcium and vitamin D replacement (Fig. 2). Magnesium replacement and discontinuation of intravenous SFO alone resulted in normalization of serum mineral balance as well as improved bone mineral density at the femoral neck (Table 2). Our case report was waived from the ethical approval or institutional review board of Hokkaido University Hospital, based upon their policy to review all intervention and observational study except for a case report. The patient provided informed consent for the publication of her clinical data. The presented data are anonymized and risk of identification is minimal.

**Table 2:**
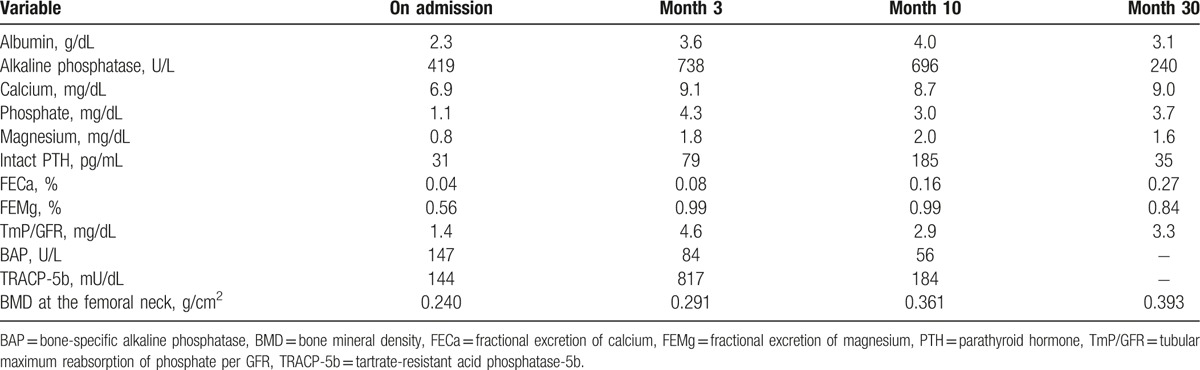
Changes of metabolic parameters before and after admission.

## 3 Discussion

Hypocalcemia can arise from insufficiencies of parathyroid hormone (PTH) and/or activated vitamin D. Because hypomagnesemia is known to be one cause of such pathophysiology,^[6]^ it is important to measure serum magnesium level in cases of prolonged hypocalcemia. Magnesium is absorbed mainly through the intestine, and serum concentration is maintained by the mediation of reabsorption and excretion by the kidney.^[7]^ Thus, decreased magnesium intake due to malnutrition or decreased uptake due to metabolic disease, malabsorption syndrome, or renal dysfunction can lead to hypomagnesemia. In the present case, fractional excretion of magnesium was quite low (0.56%), even in the presence of hypomagnesemia, and the residual intestine was very short (<1 m) because of multiple intestinal resections. Taken together, magnesium malabsorption was thought to be the main cause of hypomagnesemia in this patient.

Other hands, serum phosphate concentration is regulated through balances among absorption by the intestinal tract, excretion by the kidney, and buffering in bone metabolism. Several humoral factors, such as FGF23, 1,25-dihydroxyvitamin D (1,25(OH)_2_D), and PTH are associated with this homeostasis. In general, hypophosphatemia promotes production of 1,25(OH)_2_D and can lead to elevated serum 1,25(OH)_2_D concentration. However, FGF23 was revealed to lower serum phosphate levels by inhibiting vitamin D activation via both decreased renal mRNA expression of a metabolic enzyme of vitamin D (25(OH)D-1α-hydroxylase) and increased expression of 25(OH)D-24-hydroxylase^[8]^; resulting in inhibition of reabsorption of phosphate in the proximal tubule and bowel. Recently, it has been strongly recommended that patients with osteomalacia with hypophosphatemia have serum FGF23 level checked. Intravenous SFO administration had been reported to lead to hypophosphatemia accompanied by phosphate diabetes, especially in Japan.^[9]^ Although the precise pathogenesis of this type of hypophosphatemia has not been clarified, the relationship between elevated serum FGF23 level and SFO-induced hypophosphatemia was recently verified.^[4,5]^ Elevation of FGF23 can be caused not only by SFO, but also by intravenous iron polymaltose administration; however, other kinds of intravenous and oral iron replacements have not been shown to increase FGF23 concentration.^[10,11]^ Although the precise reason for this discrepancy has not been clarified, a previous prospective study mentioned the possibility that certain iron preparations inhibit FGF23 degradation in osteocytes, leading to a temporary elevation of intact FGF23.^[12]^ In this case, considering that interruption of SFO administration normalized serum phosphate and FGF23 levels, SFO appeared to be the main cause of the patient's FGF23 elevation. In general, vitamin D-dependent osteomalacia is treatable with appropriate vitamin D supplementation, whereas vitamin D-resistant osteomalacia, most often accompanied by FGF23 elevation, requires not only vitamin D supplementation but also phosphate replacement therapy to maintain serum phosphate levels. If SFO-induced hypophosphatemia is diagnosed, because elevated FGF23 level and hypophosphatemia are treatable,^[9]^ discontinuation of SFO alone may be sufficient in most cases. However, in cases of a long period of osteomalacia, it takes a relatively long period for serum mineral levels and vitamin D concentration to normalize,^[13]^ as in the present case. In case of such as severe bone pain due to osteomalacia, prolonged mineral imbalances or quite low 1,25(OH)_2_D level, vitamin D, and/or phosphate supplementations should also be considered. In the present case, our patient's FGF23 level was quite high (314 pg/mL) and was accompanied by low levels of 1,25(OH)_2_D, PTH, and tubular maximum reabsorption of phosphate per glomerular filtration rate in addition to malabsorption of phosphate due to short-bowel syndrome.

With regard to the hypocalcemia, multiple factors might be involved: malabsorption of calcium from the bowel, PTH dysfunction due to hypomagnesemia, inhibition of vitamin D, and degradation of 1,25(OH)_2_D production due to PTH insufficiency, and elevation of FGF23.^[8]^ It has been demonstrated that the activity of adenylate cyclase is inhibited, as well as renal and skeletal resistance to PTH action is increased under in the hypomagnesemic state.^[14]^ In this case, serum 25-hydroxyvitamin D (25(OH)D) was undetectable in spite of vitamin D supplement. SFO-induced hypophosphatemia does not always represent 25(OH)D deficiency. Malabsorption of vitamin D due to short-bowel syndrome, a situation that would lead to exacerbation of the deranged mineral balance, would exist. Overall, the multiple mineral imbalances and humoral factors resulting from both inappropriate SFO administration and short-bowel syndrome in the present case could have led to refractory hypocalcemia and severe osteomalacia.

In conclusion, we have reported a case of complicated mineral imbalance and osteomalacia that were effectively treated with magnesium replacement and interruption of SFO administration. It is important to avoid inappropriate SFO administration and to perform a careful evaluation of minerals, including phosphate and magnesium, especially in patients with short-bowel syndrome.
